# Comparisons of Three Methods for Organic and Inorganic Carbon in Calcareous Soils of Northwestern China

**DOI:** 10.1371/journal.pone.0044334

**Published:** 2012-08-31

**Authors:** Xiujun Wang, Jiaping Wang, Juan Zhang

**Affiliations:** 1 State Key Laboratory of Desert and Oasis Ecology, Xinjiang Institute of Ecology and Geography, Chinese Academy of Sciences, Urumqi, Xinjiang, China; 2 Earth System Science Interdisciplinary Center, University of Maryland, College Park, Maryland, United States of America; 3 Graduate University of Chinese Academy of Sciences, Beijing, China; University of Delaware, United States of America

## Abstract

With increasing interest in the carbon cycle on arid land, there is an urgent need to quantify both soil organic carbon (SOC) and inorganic carbon (SIC) thus to assess various methods. Here, we present a study employing three methods for determinations of SOC and SIC in the Yanqi Basin of northwest China. We use an elemental analyzer for both SOC and SIC, the Walkley-Black method for SOC, a modified pressure calcimeter method for SIC, and a simple loss-on-ignition (LOI) procedure for determinations of SOC and SIC. Our analyses show that all three approaches produce consistently low values for SOC (1–14 g kg^−1^) and high values for SIC (8–53 g kg^−1^). The Walkley-Black method provides an accurate estimate of SOC with 100% recovery for most soil samples. The pressure calcimeter method is as accurate as the elemental analysis for measuring SIC. In addition, SOC and SIC can be accurately estimated using a two-step LOI approach, i.e., (1) combustion at 375°C for 17 hours to estimate SOC, and (2) subsequent combustion at 800°C for 12 hours to estimate SIC. There are strong linear relationships for both SOC and SIC between the elemental analysis and LOI method, which demonstrates the capability of the two-step LOI technique for estimating SOC and SIC in this arid region.

## Introduction

Soil carbon storage, as the third largest carbon pool in the Earth System, plays an important role in the global carbon cycle and climate change [1]. The majority of carbon, in most soils, is held as soil organic carbon (SOC) whereas in soils of the arid and semiarid regions, the most common form is inorganic carbon, primarily carbonate [2]. Quantifying both SOC and soil inorganic carbon (SIC) is essential to our understanding of the carbon cycle at regional to global scales.

Soil organic carbon is commonly measured by dry combustion with automated analyzers, or a wet chemical oxidation method, i.e., the Walkley and Black method [3]. The automated technique is simple and accurate, but the cost is high and may not be feasible. In addition, this approach often involves pretreatment with acid to remove carbonate for calcareous soils, which may erode the instrument and destroy organic matter in soil samples [4]. On the other hand, the Walkley and Black method has been used as one of the standard methods to determine SOC [5], particularly in China [6]. This technique is less expensive than dry combustion. However, this procedure may lead to variable recovery of SOC [7], [8], and hazard Cr due to the use of dichromate.

Soil inorganic carbon can be accurately measured by several methods [9]. Commonly, SIC is determined by measuring CO_2_ production after adding HCl acid into soil [10]–[13]. Sherrod et al. [12] demonstrated that a modified pressure calcimeter method, with addition of FeCl_2_ to minimize oxidation of organic matter, provided reliable measurement of SIC. Alternatively, dry combustion with automated analyzers can also be utilized to determine SIC. A direct measurement involves dry combustion of soils that are pre-combusted to remove organic matter in an O_2_ stream [14]. An indirect measurement is to determine total carbon (without pretreatment with acid to remove carbonate) and SOC separately, then to calculate the difference. Although the automated approaches would provide accurate estimates for SIC, they might not be used widely because of the high costs associated with purchase and maintenance of automated analyzers.

Apart from these traditional methods, loss-on-ignition (LOI) provides an alternative approach, which involves heating soil samples at high temperature to combust soil organic matter (SOM) or carbonate and measuring weight losses [15], [16]. The LOI techniques are simple, less expensive and less labor intensive relative to the automated techniques and chemical methods. Thus, the LOI approaches have been used widely to estimate SOM or SOC in agricultural and forest soils and sediments [16]–[21], and to determine carbonate content in sediments [13], [15], [22], [23]. However, this technique is rarely applied to low fertility soils for SOM or SOC measurement, or used to measure SIC despite some studies showing that the LOI method can give accurate estimates of carbonate for sediments [13], [24]. Moreover, there is little utilization of the LOI methods for estimating SOC and SIC in Chinese soils.

The arid and semiarid lands cover approximately 35% of the Earth’s land surface, and may play an important role in the global carbon cycle and climate mitigation [1], [25], [26]. With recent interest in studying the carbon cycle on arid land [27], there is a need to quantify both SOC and SIC of arid soils thus to assess various methods. Here, we select three techniques to determine SOC and SIC contents in the calcareous soils at the Yanqi Basin of northwest China. We employ an elemental analyzer for both SOC and SIC measurements, a wet oxidation for SOC, a modified pressure calcimeter method for SIC, and a simple LOI procedure for determinations of SOC and SIC. The objective of this study is to examine the relationships between these methods, and to test the hypothesis that the LOI technique has capacity of accurately determining SOC and SIC in arid soils.

## Materials and Methods

### Site Description

**Figure 1:**
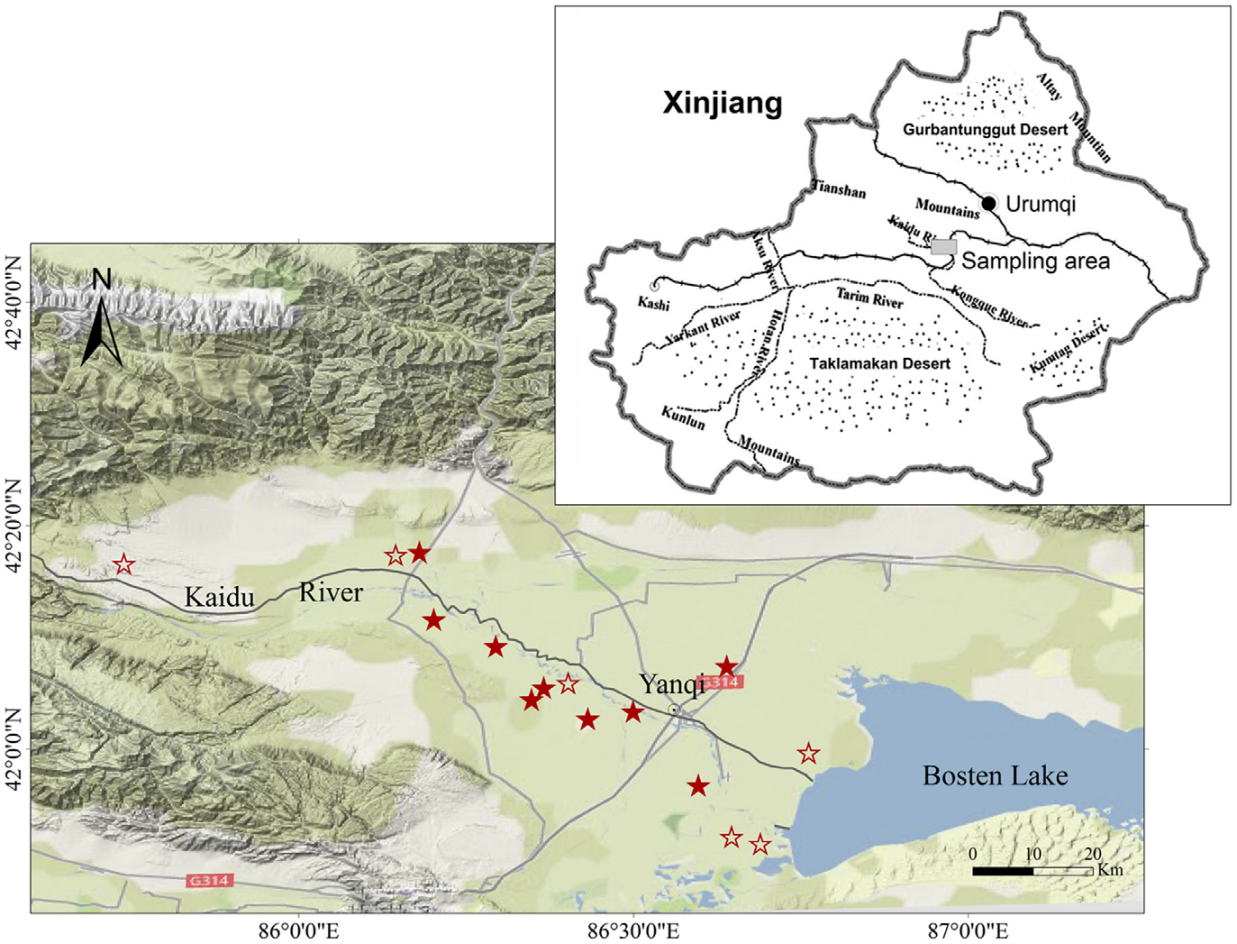
Sampling area with locations of 15 soil profiles. Solid stars denote the sites where topsoil texture was determined.

There were no specific permits required for the described field studies. We confirm that the location was not privately-owned or protected in any way, and the field studies did not involve endangered or protected species. Our studying area is along the Kaidu River in the Yanqi Basin (Figure 1), which is located on the southeastern flank of the Tianshan Mountain. Brown desert soil is the main soil type, which was developed from limestone parent material and classified as a Haplic Calcisol [28]. The sampling area (approximately 2,200 km) spans both sides of the Kaidu River, which has various land uses, including desert land, shrub land, cropland and grassland. The area has an annual precipitation of less than100 mm and annual evaporation of approximately 2000 mm. Agricultural lands have irrigation systems that extract water from the Kaidu River, whereas the other lands rely on rainfall and underground waters. Annual mean temperature ranges from 7°C to 10°C. On average, the annual maximum temperature is 38°C, and the minimum temperature −35°C.

### Soil Sampling and Analyses

We collected 70 soil samples from 15 profiles in November, 2010. While these profiles vary in texture and color because of land use history, we sampled five layers at most profiles, i.e., 0–5 cm, 5–15 cm, 15–30 cm, 30–50 cm, and 50–100 cm, and determined bulk density (BD). For each layer, soils were air-dried, thoroughly mixed, and sieved to pass a 2-mm screen for pH and electric conductivity (EC) measurements. Basic soil properties are given in Table 1. In brief, soils in this region are characterized by high pH (from 8 to 9.4) and high sand/silt contents. Bulk density is slightly lower in the surface soils (0–15 cm: 1.38–1.41 g cm^−3^) than in below (1.46–1.54 g cm^−3^). Electric conductivity shows a decreasing trend over depth, from 1.25–1.32 ms cm^−1^ in the 0–15 cm to 0.7 ms cm^−1^ in the 50–100 cm. Representative sub-samples were crushed to pass a 0.25 mm screen for SOC and SIC measurements by the following procedures.

**Table 1:** Means and standard deviations (in brackets) of soil pH, bulk density (BD, g kg^−1^) and electrical conductivity (EC, ms cm^−1^) for each layer, and clay, silt and sand contents (%) of topsoil (0–30 cm) from 8 randomly selected soil profiles.

	pH	BD	EC	Clay	Silt	Sand
0–5	8.92 (0.3)	1.41 (0.1)	1.25 (2.4)	5.6 (2.9)	57.3 (23.0)	37.1 (25.1)
5–15	8.77 (0.3)	1.38 (0.1)	1.32 (1.5)			
15–30	8.78 (0.4)	1.50 (0.2)	1.07 (1.2)			
30–50	8.88 (0.4)	1.54 (0.1)	0.98 (1.2)	–	–	–
50–100	9.00 (0.2)	1.46 (0.1)	0.70 (1.2)	–	–	–

### Modified Walkley-Black Method for SOC

This method is modified from the traditional Walkley-Black method [3]. A soil sample (∼0.4 g) is treated with 5 ml concentrated H_2_SO_4_ for 4 hours, then mixed with 5 ml 0.5 M K_2_Cr_2_O_7._ The mixture is heated at 150–160°C for 5 minutes, and then cooled at room temperature. The solution is transferred into a triangular flask with 100 ml deionized water. Unreacted K_2_Cr_2_O_7_ is determined by titrating with 0.25 M FeSO_4_. Soil organic carbon content is calculated, without a recovery factor, from the difference in FeSO_4_ used between a blank and a soil solution.

### Pressure Calcimeter Method for SIC

We use a procedure similar to that of Sherrod [12]. A subsample of 1.0 g soil and a tiny bottle with 2 ml 6 M HCl containing 3% by weight of FeCl_2_·4H_2_O are placed at the bottom of a 100-ml reaction vessel. After sealing the vessel with a butyl rubber stopper that has an aluminum tearoff seal cap, we mix the acid with soil by turning the vessel sideways. Two hours later, we remove the aluminum tearoff seal cap, and then insert an 18-gauge hypodermic needle that is attached to the pressure transducer and voltage meter. We record the voltage reading to two decimal places after 3 to 5 seconds, and then calculate CO_2_ concentration using a calibration curve that is developed by mixing reagent grade CaCO_3_ with oven-dried laboratory sand. Standards are made based on a final weight of 1.0000 g mixture of sand and CaCO_3_ to obtain inorganic carbon concentrations of 1.2, 3.6, 6.0, 12.0, 18.0, 24.0, 30.0, 36.0, 42.0, 48.0, 54.0 and 60.0 g C kg^−1^, respectively.

### Elemental Analyzer for SOC and SIC

We used a CNHS-O analyzer (EuroEA3000) for SOC and SIC estimates. For SOC measurement, 20 mg soil is pretreated with 10 drips of H_3_PO_4_ for 12 hours to remove carbonate. The sample is combusted at 1020°C with constant helium flow carrying pure oxygen to ensure complete oxidation of organic materials. CO_2_ production is determined by a thermal conductivity detector. Total soil carbon is measured, using the same procedure without pretreatment with H_3_PO_4_. Soil inorganic carbon is calculated as the difference between total soil carbon and SOC.

### Loss-on-ignition (LOI) Procedure for SOM and SIC

Following Wang et al. [16] and Wang et al. [15], we first place 5.0000 g soil in a quartz glass and heated at 105°C for 12 hours to remove soil moisture. We then combust soil in a programmable muffle furnace (S1849, KOYO LINDBEERE LTD) at 375°C for 17 hours and 800°C for 12 hours. Soil organic matter is calculated as the weight loss between 105°C and 375°C, and SIC as the weight loss between 375°C and 800°C:
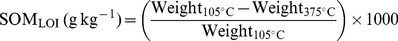
(1)

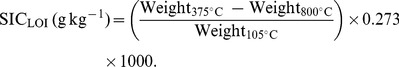
(2)


The conversion constant of 0.273 in equation (2) is applied to convert mass of CO_2_ to mass of carbon.

## Results

### Soil Organic Carbon

**Figure 2:**
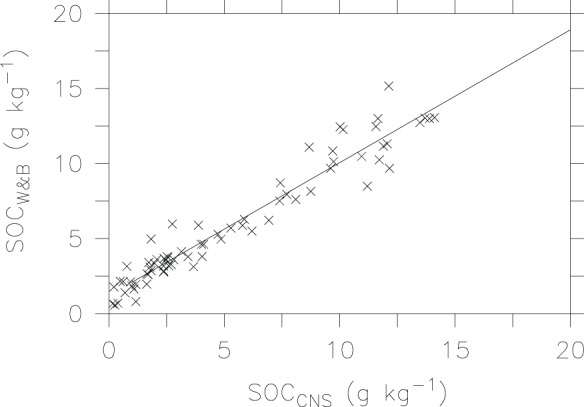
Linear regression of measured SOC by the CNS analyzer and Walkley-Black method.

We first compare measured SOC between the automated CNS analysis and the modified Walkley-Black method, and find a strong correlation (r^2^ = 0.93, P<0.001) between the two methods (Table 2). As shown in Figure 2, SOC content is below 15 g kg^−1^ in this region. It appears that the modified Walkley-Black method over-estimates SOC when soil contains extremely low SOC (<5 g kg^−1^). Excluding these data yields an averaged recovery of 0.993. These results indicate that almost 100% SOC in the brown desert soil can be oxidized, which is much higher than previous published recovery rates (less than 82%) for forest soils across Flanders region [7], but in agreement with those (∼100%) in most Tasmanian soils [16]. The high recovery of SOC and large correlation coefficient in our study suggest that the modified Walkley-Black method is as accurate as the automated CNS technique except for extremely low SOC soils.

**Table 2:** Relationships of soil organic carbon (SOC) and inorganic carbon (SIC) among different methods.

	n	Slope	Intercept	r^2^	P *value*
SOC_W&B_ vs. SOC_CNS_	70	0.886	1.199	0.92	<0.001
SOM_LOI_ vs. SOC_CNS_	70	1.792	4.189	0.90	<0.001
SOC_LOI_ vs. SOC_W&B_	31	0.949	0.443	0.77	<0.001
SIC_PC_ vs. SIC_CNS_	70	1.025	−1.718	0.99	<0.001
SIC_LOI_ vs. SIC_CNS_	70	1.043	2.582	0.96	<0.001
SIC_LOI_ vs. SIC_PC_	70	1.021	4.241	0.98	<0.001

**Figure 3:**
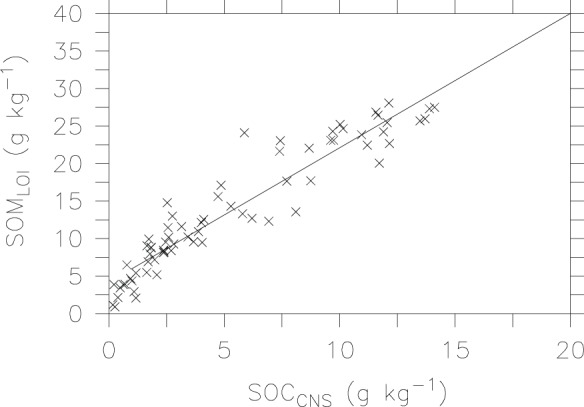
Relationship between SOC measured by the CNS analyzer and SOM determined by the LOI method.

We then compare measured SOM by the LOI method with SOC by the automated CNS technique (Figure 3). As expected, SOM values (0–27 g kg^−1^) are higher than SOC values (<15 g kg^−1^). The large intercept value of 4.189 indicates that there are some non-SOM weight losses (e.g., from structural water and carbonate) during ignition. The conversion factor of 1.792 is slightly larger than the traditional values of 1.724. We find that the conversion factor is 2.71 for those samples with low SOC content (<5 g kg^−1^) that are mainly subsoils. Based on all 70 soil samples tested, SOC may be calculated from LOI at 375°C for 17 hours, using the following equation:
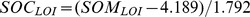
(3)


**Figure 4:**
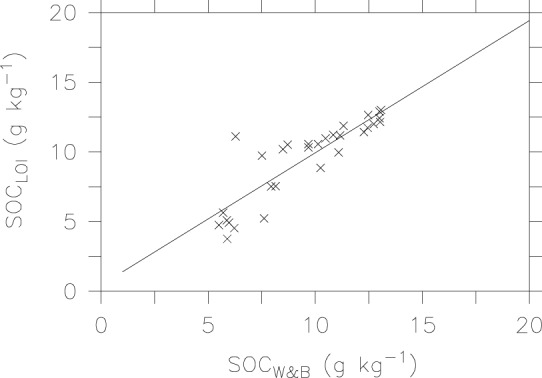
Relationship between SOC calculated by the LOI method and SOC measured by the Walkley-Black method.

Figure 4 shows comparison of measured SOC by the Walkley-Black method and calculated SOC by the LOI method using the equation (3). To avoid negative values in calculated SOC, we exclude samples that contain less than 4.189 g kg^−1^ SOM_LOI_ in this comparison. The slope value of 0.949 and significantly high correlation coefficient (r^2^ = 0.77, p<0.001) indicate that the LOI method has potential for estimating SOC in this arid region.

### Soil Inorganic Carbon

**Figure 5:**
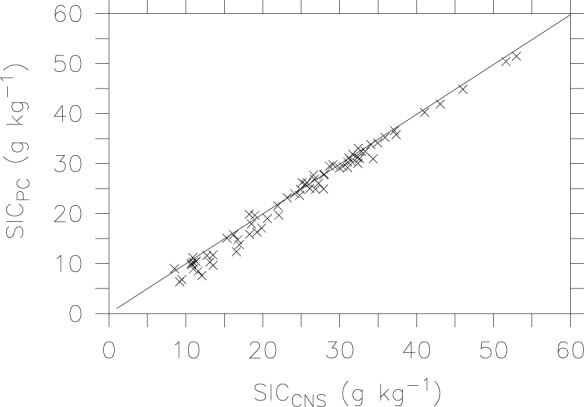
Liner relationship for SIC by the CNS analyzer and pressure calcimeter method.

We assess measured SIC by the pressure calcimeter method and the automated CNS analysis (Figure 5). Clearly, SIC content is much higher than SOC (<15 g kg^−1^) in this region. The pressure calcimeter method produces slightly lower SIC values (6–51 g kg^−1^) than the automated CNS technique (9–53 g kg^−1^). However, there is a high correlation (r^2^ = 0.99, p<0.001) between the two methods. The slope value of 0.996 and large correlation coefficient indicate that the pressure calcimeter method is as accurate as the automated CNS technique for the calcareous soils.

**Figure 6:**
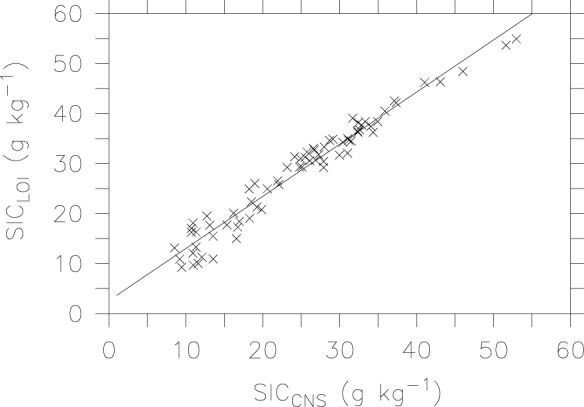
Liner relationship for SIC between the CNS analyzer and LOI method.

Figure 6 presents comparison of measured SIC between the LOI method and the automated CNS analysis. Both methods give a very similar range, and form a linear relationship:
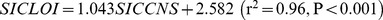
(4)


The intercept value of 2.582 is significantly different from 0, suggesting that there may be some non-SIC weight losses (e.g., dehydration from other compounds) during combustion [22], [23], [29]. Nevertheless, the factors of slope value close to 1 and significant correlation indicate that the LOI technique can produce accurate estimates for SIC.

**Figure 7:**
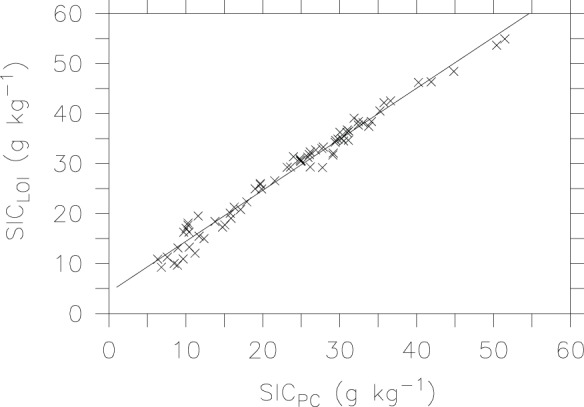
Liner relationship for SIC by the pressure calcimeter and LOI methods.

As shown in Figure 7, there is also a linear relationship for SIC between the pressure calcimeter method and the LOI technique:
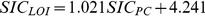
(5)which shows a similar slope to that in the regression between the LOI method and the CNS analysis (i.e., equation 4). The relatively larger intercept in equation (5) reflects the slightly lower SIC values by the pressure calcimeter method, relative to those by the automated CNS analysis (see Table 2).

## Discussion

### Estimate of SOC

Measurement of SOC has been a common procedure in soil science. Many methods are available, each with advantages and disadvantages in terms of accuracy, expense and convenience [5], [30]. The Walkley-Black procedure is considered to be cheap and easy to perform, but may exhibit variable recovery thus a correction factor is often applied to determine the total SOC content for a soil sample. For example, Sleutel et al. [31] applied a correction factor of 1.33 for estimated SOC by the Walkley-Black without external heating. However, an early study [16] showed that without external heating, the Walkley-Black method produced approximately 100% recovery for SOC in most Tasmanian soils. Many modified dichromate oxidation techniques that involve external heating do not require a correction factor [32]. Our study demonstrates that compared with the automated CNS technique, the modified Walkley-Black method with external heating yields almost 100% recovery for SOC in the calcareous soils we tested. However, the Walkley-Black method may over-estimate SOC in soils containing extremely low SOC [8], which is probably attributed to reactions of K_2_Cr_2_O_7_ with inorganic soil constituents [33].

While the LOI method has been widely used to estimate SOM and SOC, there is no standard procedure in terms of heating duration and temperature. There have been various combinations with temperature ranging from 150°C to 900°C and duration from 2 hours to 17 hours in the literature, thus a regression between LOI and SOC (measured by an automated analyzer or the Walkley-Black method) varies widely [16], [34], [35]. In general, with increasing temperature, LOI increases whereas correlation coefficient tends to decrease [35].

One criticism of the LOI technique is that it may result in weight losses from structural water and carbonate during heating [30], [34]. These non-SOM weight losses can significantly affect estimates of SOC, particularly in soils containing low SOM and high clay content. As the amount of SOM decreases, dehydroxylation of clays becomes more likely [36]. However, clay content is low in our soils (see Table 1). Thus, weight loss due to dehydroxylation of clays may be small. On the other hand, temperature is the dominant factor affecting LOI, and its relationship with SOC [35]. Therefore, it is critical to choose a temperature that is high enough to completely remove SOM but low enough to prevent dehydroxylation of clay minerals and oxidation of carbonate.

Several studies have shown that a combination of 375°C and 16–17 hours results in a good LOI-SOC relationship, thus it can provide accurate estimates of SOC in many soil groups [16], [37], [38]. Our study demonstrates that there is a strong relationship between SOC and LOI at 375°C for 17 hours in the calcareous soils tested. While the non-zero intercept may reflect some non-SOM weight losses, the strong relationship indicates the capacity of using the LOI method for SOC estimation.

### Estimate of SIC

Soil carbonate is usually quantified by acid dissolution in the reaction below:
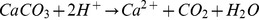
(6)with the determination of either H^+^ consumption or CO_2_ production. The methods of H^+^ consumption that involve reaction with a strong acid, such as HCl addition and back titration of the unreacted acid, may be problematic owing to H^+^ consumption by other soil components [9]. Indeed, our own analyses using the H^+^ consumption approach lead to significant underestimates of SIC (by 20%) for the soils we tested (data not shown).

There have been many methods involving determination of CO_2_ production following acid dissolution [9]. While these methods are much preferred, precautions must be taken to ensure that there is no interference from organic matter oxidation. Thus, an oxidation inhibitor (e.g., FeCl_2_) is often used to limit the oxidation of organic matter and the subsequent evolution of CO_2_ from this source. Our study shows a strong correlation between the pressure calcimeter method and the automated CNS analysis. Given that measured SIC contents by the pressure calcimeter method are slightly lower than, but not significantly different from those by the automated CNS analysis, we conclude that the pressure calcimeter approach can provide an accurate measurement of SIC for the calcareous soils of this arid region.

In contrast to wide utilization of the LOI method for SOC estimates, there is little in the literature regarding application of the LOI method for SIC estimates. However, the LOI method has been used to measure carbonate content in sediments, following combustion of organic carbon at lower temperature [13], [15], [22], [23]. The simple two-step LOI procedure for estimates of organic carbon and carbonate in sediments [15] is similar to that for determination of organic carbon and carbonate in calcareous soils by dry combustion [14]. Thus, it could be a convenient practice for estimating SOC and SIC.

To the best of our knowledge, this study is the first attempt to evaluate the LOI method for SIC measurement. Our study shows a significantly (r^2^>0.96, P<0.001) linear relationship between estimated SIC by the LOI method and determined SIC by direct measurements, i.e., the automated element analyzer and the pressure calcimeter method. Despite the non-zero intercepts and slightly higher values in SIC by the LOI method, the slope values are close to one, which are consistent with those reported for sediment analyses [15]. Our results demonstrate that the LOI method has potential for easily and accurately estimating SIC in calcareous soils of arid regions.

### Conclusions

We have compared three methods for determinations of SOC and SIC concentrations in calcareous soils of the Yanqi Basin. In spite of wide ranges in soil properties, SOC is generally low (1–14 g kg^−1^) whereas SIC is considerably high (9–53 g kg^−1^). Our study shows that the Walkley-Black method with external heating can provide accurate estimates of SOC (with almost 100% recovery) for most arid soils. The pressure calcimeter method is as accurate as the automated elemental technique for measuring SIC. In addition to these traditional methods, we have also evaluated a two-step LOI approach, i.e., (1) combustion at 375°C for 17 hours to estimate SOC, and (2) subsequent combustion at 800°C for 12 hours to estimate SIC. Our study shows that SOC and SIC can be estimated from LOI for calcareous soils except those with extremely low SOC or SIC contents. This study demonstrates that the two-step LOI technique has potential to be used as a sufficiently accurate technique for estimating SOC and SIC in arid soils. Further testing on a wider range of arid and semi-arid soils is warranted.
